# Organic–inorganic interfaces and spiral growth in nacre

**DOI:** 10.1098/rsif.2008.0316

**Published:** 2008-08-26

**Authors:** Nan Yao, Alexander K. Epstein, Wendy W. Liu, Franz Sauer, Ning Yang

**Affiliations:** 1 Princeton Institute for the Science and Technology of Materials, Princeton University70 Prospect Avenue, Princeton, NJ 08544, USA; 2 Bruker AXS Inc., 5465 E. Cheryl Parkway, Madison, WI 53711, USA

**Keywords:** nacre, biomineralization, crystal growth

## Abstract

Nacre, the crown jewel of natural materials, has been extensively studied owing to its remarkable physical properties for over 160 years. Yet, the precise structural features governing its extraordinary strength and its growth mechanism remain elusive. In this paper, we present a series of observations pertaining to the red abalone (*Haliotis rufescens*) shell's organic–inorganic interface, organic interlayer morphology and properties, large-area crystal domain orientations and nacre growth. In particular, we describe unique lateral nano-growths and paired screw dislocations in the aragonite layers, and demonstrate that the organic material sandwiched between aragonite platelets consists of multiple organic layers of varying nano-mechanical resilience. Based on these novel observations and analysis, we propose a spiral growth model that accounts for both [001] vertical propagation via helices that surround numerous screw dislocation cores and simultaneous 〈010〉 lateral growth of aragonite sheet structure. These new findings may aid in creating novel organic–inorganic micro/nano composites through synthetic or biomineralization pathways.

Biomaterials provide fascinating examples of nature's ability to assemble structures of remarkable strength and toughness (Aksay *et al*. 1996; Addadi & Weiner 1997; Sanchez *et al*. 2005). Particularly well studied is nacre, or mother of pearl, a composite of aragonite CaCO_3_ and organic matrix that is 3000 times more fracture resistant than the pure mineral (Currey 1977; Jackson *et al*. 1988). Its unique mechanical properties are attributed to a structure of polygonal aragonite platelets, approximately 5 μm across by 0.5 μm thick, wrapped in polysaccharide and protein fibres (Weiner 1986; Jackson *et al*. 1989). There exist two principal types of nacre structure: columnar, composed of stacked platelets of rather uniform size with coinciding centres, and sheet, in which the centres of platelets rest on the interfaces between underlying platelets, as in a brick wall. Columnar nacre is known to be deposited in a narrow zone at the margin of the shell, while sheet nacre is deposited over most of the inner surface (Hedegaard 1997). Yet, after 160 years of scientific investigation (Carpenter 1847), the precise features governing nacre's extraordinary strength remain elusive. This is largely due to the lack of structural characterization at the atomic and nanometre scale. Understanding the physical and mechanical properties of nacre at nanometre scale dimensions could lead to the synthesis of novel materials with unprecedented performance.

Here, we present a series of observations, using high-resolution imaging and nanoscale force measurements, on structural features at the organic–inorganic interface, morphological and mechanical properties of the organic matrix, crystal directions and ultimately sheet nacre growth. We reveal nanometre-scale growths on vertical (010) platelet faces and new details of asperities, i.e. small protrusions, on horizontal (001) faces of aragonite platelets in the red abalone shell (*Haliotis rufescens*). Analysis of (001) platelet surfaces showed groove-like imprints from nano-asperities in the overlying layer. The remarkable alignment of these asperities, which corresponds to the crystal orientation of the aragonite, indicates that large domains of aragonite platelets within lamellae share the same orientation. We further demonstrate the platelet surface properties and organic layers surrounding the platelets and reveal distinct differences in the molecular morphology and adhesive properties of each organic layer. Finally, we incorporate these novel findings into a spiral growth model for gastropod nacre formation. This spiral growth model accounts for both lateral growth of the aragonite lamellae and [001] vertical propagation via helices that surround numerous screw dislocation cores.

There has been a range of theories proposed to explain the growth mechanism of nacre (Watabe 1965, 1981; Beveland & Nakahara 1969; Nakahara 1979; Weiner & Traub 1980; Morse *et al*. 1993; Schaffer 1997; Cartwright & Checa 2007). A widely discussed model proposes that aragonite layers grow in the [001] stacking direction via mineral bridges that penetrate the organic matrix (Schaffer 1997). However, previous studies have failed to explicitly document such mineral connections at the atomic lattice level, which is the key to demonstrating crystalline continuity through the bridge. Here, we use high-resolution transmission electron microscopy (HRTEM) to examine these mineral bridges of sheet nacre and find that in all observed cases, they are not physically connected as a single crystal. As shown in figure 1, nanoscale growth structures were observed on both vertical (010) and horizontal (001) faces of aragonite platelets. Atomic lattice structure in the vertical interface region marked with dashed circles in figure 1*a* is detailed in figure 1*b*–*d*, where figure 1*b* shows the shoulder region of the platelet and figure 1*c* gives a selected-area diffraction pattern from the lower circled region in figure 1*a* demonstrating that the asperities and the platelet are of the same crystal orientation. The pyramidal nano-growths of figure 1*d* are oriented in the [010] direction. They somewhat resemble the larger nano-asperities observed on the horizontal (001) faces of the platelets as shown in figure 1*e*–*i*. However, they exhibit a larger aspect ratio and appear to extend approximately 10–15 nm from the (010) face. Lattice imaging of [001] growth structures shows that these nano-asperities, even when they coincide and appear to be collinear, do not physically connect the platelets, but are merely interlocking. As shown in figure 1*e*–*h*, these ‘mineral bridges’ are formed by the merging of pyramids from both top and bottom platelets. They do not fully traverse the organic matrix, and the two platelets are not epitaxial as one single crystal. It may be conjectured that slightly varying the depth of the image cross section would produce contrary evidence. Yet, while the images in figure 1 cannot categorically disprove the existence of [001] mineral bridges in both columnar and sheet nacre structures, the images clearly show that in a typical cross section even the most seemingly connected asperities are discontinuous. If mineral bridges were ubiquitous structures, as has been suggested, one would expect to see a clear indication of crystalline continuity in such a cross section. Instead, the HRTEM analysis shows nearby but separate asperities associated with neighbouring platelets in the [001] stacking direction. Figure 1*e*,*f* features the largest overlap between asperities, yet a close inspection of the contact region clearly reveals a horizontal domain boundary with a crystalline discontinuity and minimal lattice coherence, ruling out a continuous single crystal. It should again be emphasized that images of ‘columns’ between platelets previously cited as evidence for the existence of mineral bridges have been at lower resolutions, as those arrowed in figure 1*a*,*i*; thus, they were unable to resolve at atomic scale the two separate nano-asperities that together appear to form bridges.

The nano-asperities, although they may not connect platelets across the organic layer, do share the crystal orientation of their host aragonite platelet as demonstrated in the images and diffraction pattern in figure 1. Depending on the nature of the organic layers remaining on the sample surface after cleavage of the nacre, the groove-like imprints of asperities from the removed overlying layer can be visible via scanning electron microscopy (SEM). Large areas of several nacre samples are examined where these imprints appeared, and it is found in figure 2*a*,*b* that the asperities displayed a high degree of alignment. All asperities grown on the same platelet are aligned along the same direction (Wang *et al*. 2001), and the remarkable alignment of those asperities over large areas on the (001) plane indicates that these platelet domains share the same orientation. Since the aragonite platelets are of orthorhombic (*pmcn, No. 62*) structure with their [001] axes aligned along the stacking direction, these images indicate that asperities prefer three lateral crystal orientations, roughly 120° apart.

These domains of identically oriented platelets within lamellae imply that the crystal direction has been transmitted laterally, in the 〈010〉 or 〈110〉 directions. Most previous growth models have proposed the dominant growth to be in the [001] direction, as this is the direction in which the nacre growth front propagates, and they have suggested no physical connection or certain relationship in crystal orientation between platelets in the lateral direction. A notable exception was the early hypothesis of Wada (1966) that ‘nacre grows by the advance of growth fronts of mineral terraces which begin from deformed, misfitting or boundary portions on the nacreous surface’; however, the study could not explore the mechanisms of any such growth since evidence from high-resolution imaging was not available. The spiral growth model we propose here accounts for both lateral growth of the aragonite lamellae and [001] propagation via helices that surround numerous screw dislocation cores, which systemically pervade abalone nacre. The relationship between crystallographic directions and corresponding surface structures on a core platelet are shown in figure 3.

We have observed pervasive screw dislocation cores using SEM over a large surface area, as shown in figure 4 where 30 such cores are visible. An average screw dislocation density of 10^5^ cm^–2^ is found in this study, which suggests that each screw dislocation core is associated with approximately 30–40 platelets. Further analysis shows that these screw dislocations are associated with the surface steps and many, if not all, of them are paired, i.e. the growth step from a right-handed screw dislocation core is shared by a nearby left-handed core, or vice versa. The growth step exists as a finite line defect delimited by the two screw dislocation cores of opposite sign. The separation of paired cores could range from less than 20 to over 100 μm. Among 30 cores 22 are visibly paired, while the remaining 8 cores have pairs just off the screen. Based on these findings, the spiral growth model can account for paired screw dislocations that result in [001] nacre growth via connected growth steps throughout the nacre. Different stages of the growth step as it moves relative to the two cores can be seen clearly in images on the right-hand side of figure 4: a convex growth step that is diverging past the cores (a–a), and three concave steps that are converging between the cores (b–b, q–q and r–r). These screw dislocation cores have been reported to exhibit zigzag tessellations, or jogs, as they pass through multiple aragonite layers (Yao *et al*. 2006). The crystal orientations of platelets in the vicinity of screw dislocation cores are aligned, whereas all three preferred orientations, approximately 120° apart, begin to appear farther from the cores. This is consistent with our model in which spiral domains extend outward from the cores and intersect in the outlying regions.

In order to confirm the observation of a long-range lateral orientation alignment in the aragonite platelets as discussed above, we have used the X-ray diffraction method (Andonov 1991) to examine a similar region over 200 μm^2^ in size. Figure 5*a* shows a raw data frame of the aragonite (002) and (012) reflections, which indicates a strong preferred orientation alignment. The gamma angle between the two reflections is approximately 18°, which is in good agreement with the orthorhombic structure of aragonite. The aragonite (012) pole figures are measured with different incident angles at 5° and 10°, respectively, as shown in figure 5*b*,*c*. The 5° incident beam penetrates to 5 μm below the sample surface while the 10° incidence covers a depth of approximately 11 μm deep under the surface layers. It shows clearly that the two pole figures are very similar, suggesting a preferred in-plane orientation not only in the top platelet layer, but also down to a depth of more than 20 layers. A simulation of these pole figures gives a spread width of 7.8° for a 5° incident angle and 7.9° for the 10° incidence. This strongly indicates that the orientation of aragonite platelets over a large area remains unchanged as a function of depth.

It is observed that some of the organic layers remained intact on areas of the nacre surface following cleavage, leading to several novel results. On certain areas, the organic matrix from the (010) or {110} faces of the removed top aragonite platelets remained; as seen in both SEM image (figure 6*a*) and atomic force microscopy (AFM) image (figure 6*b*), they indicate the honeycomb-like outlines of the removed platelets superimposed on the exposed layer, effectively providing surface information of two adjacent layers at once. These images reveal that platelets in adjacent layers along the stacking direction do not reside on top of one another but overlap extensively, showing the characteristic of sheet nacre structure. In fact, the typical number of platelets on the top layer overlapping with a given platelet on the lower layer is between three and six. Since these adjoining platelets can conceivably exhibit any combination of the three preferred crystal orientations in the lateral direction, yet many individual platelets express only one orientation as discussed above, it suggests that the lamellar layers of aragonite propagate laterally, possibly via the observed nano-growths along one of three crystal orientations normal to [001], such as [010], [−1,−1,0] or [1,−1,0]. Explicit image-based evidence of nano-growth continuity between platelets has not yet been obtained. It is much more difficult to image continuous 〈010〉 nano-growths than any continuous [001] asperities, since they are much narrower: approximately 15 nm at their base compared with approximately 50 nm. It is currently unclear why these nano-growths are smaller and narrower than their asperity counterparts, though this might be related to the preference of the crystal growth orientation. One possibility may be that such an aspect ratio, along with the variation of molecule morphology between (010) and (001) interfaces, facilitates their penetration of the organic matrix and allows them to reach the neighbouring platelet wall in the 〈010〉 direction. This would be supported by our findings regarding differences in the physical and mechanical properties of the (001) and (010) organic matrices as discussed below.

We further investigated the organic layer morphology and adhesive properties using contact mode AFM (Magonov & Yerina 2005), as shown in figure 6*b*–*e*. Surface layers of cleaved nacre were peeled to expose the underlying organic matrix. The fresh organic surface presents a pattern of densely packed globular molecules, which are probably proteins (Blank *et al*. 2003). The (001) organic interlayer and the (010) vertical organic matrix between adjacent platelets displayed significant differences. Similar to the SEM image in figure 6*a*, figure 6*b* shows that the organic matrix between the (010) faces of removed platelets resembles a honeycomb pattern. A high-magnification image in figure 6*c* reveals that this type of honeycomb segment consists of a chain of molecules with an average diameter of 249±26 nm. By comparison, the (001) interlayer organic matrix is multilamellar, as seen by the stacking layers of molecules covering the platelets in figure 6*d*. The molecules in this matrix are significantly smaller on average, with a diameter of 149±20 nm across the layers. Furthermore, the vertical and interlayer matrices exhibit distinct surface force and frictional properties, as shown in figure 6*e*. We measured the breaking force between the AFM cantilever and the sample surface by drawing the tip towards the surface and pulling it away upon contact. Four surface layers were identified in figure 6*d* based on the heights of the surface. One consists of the boundary of (010) organic matrix left over from the overlying layer (A), while the other three are different layers on top of the aragonite platelets within the honeycomb pattern (B–D). Twelve samples of comparable height (±5 nm) from each layer were collected and we found that each layer has a distinct breaking force, ranging from 4.2±0.2 nN at the border region (A) to 18.9±1.0 nN at the bottom layer (D). It is noteworthy that the top layer is the closest to the removed aragonite platelet surface among the multiple layers in the organic matrix. These observations yield insights into the mechanisms of nacre assembly. The differences between the organic matrices may affect the penetration of the nano-asperities on the horizontal and vertical faces of the aragonite platelets. Since the vertical organic layer is composed of larger molecules with lower adhesiveness relative to the (001) interlayer matrix, it is probably more porous, allowing for preferred [010] lateral mineral penetration during nacre growth.

The AFM imaging and force measurements suggest that each surface corresponds to distinct organic layers and that the organic matrix varies in adhesiveness according to height. This may account for the different patterns of organic film seen in the SEM images in figures 7 and 8. The organic matrix that is sandwiched between the aragonite platelets is revealed to have a layered structure with layer boundaries as indicated by arrows in figure 7*a*. As shown in figure 7*b*, three layers (I–III) ascend from bottom left to top right. Although the observation that the organic matrix is multilayered was previously made by Blank *et al*. (2003), the individual layers, as well as differences among them, have not been reported until now. According to two independent studies (Schaffer 1997; Weiss *et al*. 2002), the matrix consists of a water-insoluble chitin core sandwiched between two protein layers. Yet, our surface images show that more than this number of layers must exist: since the nano-asperity imprints in the three flat layers of figure 7*b* are all concave (i.e. negatives of the asperities from platelets above), these layers are all in the upper half of the matrix covering this area of the surface. In the bottom half of the matrix, the protruding asperities of the underlying platelet should exist, as evidenced in figure 1*a*,*i* and the images of Wang *et al*. (2001). Thus, at least four and possibly six or more film-like layers comprise the organic interlayer. Using SEM image analysis, the thickness of the top layer in figure 7*c* is measured as approximately 40 nm. This is remarkable, considering that the thickness of the entire organic interlayer in cross sections has been reported to be approximately 20–30 nm (Smith 1999). The mechanical stress from our peeling method of cleavage evidently stretched the interlayer adhesive ligaments to several times their original thickness, a possibility suggested by Smith. These layers exist throughout all strata of the nacre, as seen in figure 7*a*–*d*, and even follow screw dislocations, as seen in figure 7*e*.

Another interesting observation is that the depth of the nano-asperity imprints in the interlayer varied inversely with the height of layers present. Relative depth and concavity of the imprints could be seen from their darkness, since fewer secondary electrons are emitted from there and collected by the detector, compared to the edges of the surrounding layer. The imprints would have been expected to be shallower where the top layer or layers (which are adjacent to the asperities) are removed, but the opposite is true, as seen in figure 8*a*. The imprints on the platelets are more pronounced in areas without the top layer (right-hand side). Similarly, in figure 8*b*,*c*, the overturned platelets show that the top and bottom platelet surfaces have different layer morphologies, with imprints only appearing on one of two sides on peeled platelets. The variation of imprint depth on the different organic layers suggests that the mechanical properties of these layers must, as a result, be distinct: a higher resilience in the layer (or layers) adjacent to the platelet surface is implied, for the imprints are filled in by elastic recovery. This is consistent with our AFM force measurement, as well as the desirability of juxtaposing hard and soft materials (aragonite and proteins) in a biomineralized composite. Also, both this resilience and the expanded thickness of the organic layers following cleavage are consistent with the mechanical findings of Smith (1999) that the adhesive ligaments in nacre's organic matrix can lengthen to many times the original spacing between the platelets. Not only can the ligaments significantly stretch through repeated molecular unfolding, as when the aragonite layer above is peeled off, but also there is evidence that the opened intrachain loops or folded domains within interlayer molecules can partially refold after force is removed (Smith 1999). Such refolding may account for the observed filling in of the nano-asperity grooves.

New information about the structural features of nacre, including the 〈010〉 nanometre-scale aragonite growths on the vertical faces of aragonite platelets, organic layer morphology and adhesive properties, suggests that the crystal could propagate in the lateral directions. Analysis of platelet crystal orientation patterns, based on the directions of nano-asperity imprints as seen in figure 2, leads to a model of 〈010〉 growth around screw dislocation cores; as seen in large domains of lamellar nacre with uniform crystalline orientation, this crystallographic information has been physically transmitted between platelets in the same layer. The platelets both comprising and contiguous to screw dislocation cores uniformly display one of the three preferred crystalline orientations. As a result, it is logical to suggest that crystal orientation propagates in the [001] direction via these continuous cores, while orientations from different cores meet in the outlying areas, approximated by the walls of Voronoi cylinders.

These mechanisms and new observations are consistent with a spiral growth model of nacre as shown in figure 9, which accounts for both horizontal growth of aragonite platelets and vertical propagation via helices that surround numerous screw dislocation cores (see arrows). In this model, screw dislocation cores are surrounded by spiral growth domains: continuous aragonite helices. Crystal orientation is thus preserved from layer to layer in the nacre via continuous 〈010〉 growth between platelets. This lateral growth may be mediated by the 〈010〉 nano-growths, which may be mineral vesicles budding into the surrounding space. These vesicles would carry crystallographic information to the surrounding platelets and provide a supply of raw materials for further mineralization. The new platelets can either form by nucleation from the mineral vesicles (Nudelman *et al*. 2006) or by vesicle aggregation (Penn & Banfield 1998; Rousseau *et al*. 2005; Li *et al*. 2006). The existence of numerous nano-growths along the vertical face of the platelets suggests that the second mechanism is more likely. Our hypothesis is consistent with the observation that [001] asperities reflect the crystal orientation of their host aragonite platelets. Differences in properties between the vertical organic matrix and the horizontal organic interlayers may explain why crystal propagation is dominated by lateral growth in the 〈010〉 directions as opposed to spiral growth in the [001] direction for the sheet nacre structure. The screw dislocation cores are paired, and the growth steps are delimited by opposite-handed cores. These paired dislocations and the moving growth step are suggestive of Frank–Read sources, which in single crystals produce expanding dislocation loops. Slight drifting of the pivot points leads to jog-like shifting of the dislocation cores between layers (Yao *et al*. 2006).

Meanwhile, it appears that at least three or more layers exist in the organic ‘mortar’ between aragonite platelets in adjacent layers. Moreover, within this multilayer morphology, it is of great interest that the different organic layers show varying levels of mechanical resilience associated with molecules unfolding and refolding. These variations in the different layers may be important factors contributing to the ductility and toughness of nacre, and could suggest new principles for creating novel organic–inorganic composites through synthetic or biomineralization pathways.

## 1. Methods

### 1.1 Sample preparation

Shells from southern California red abalone (*Haliotis rufescens*) were used in this study. Nacre samples were sliced using a water-cooled, low-speed diamond saw and mechanically polished. The SEM sample was coated with 2–4 nm Ir to ensure conductivity for electron imaging. The final transmission electron microscopy (TEM) sample was prepared using a standard ion-milling technique with a 3 keV argon beam at an inclination angle of 12°. AFM samples from which surface layers were peeled were held with precision tweezers while a strip of adhesive Scotch tape was used to remove the layers.

### 1.2 Microscopy and X-ray diffraction

SEM images were collected on a FEI Strata DB 235 DualBeam SEM/FIB system operated at 5 kV. The TEM images were obtained using a Philips CM200 field-emission gun TEM instrument operated at 200 kV, and AFM experiments were performed using a DI Nanoscope IIIa atomic force microscope with a NanoScope V Control Station. The X-ray diffraction data were collected on a Bruker D8 DISCOVER with GADDS. The Cu-Kα X-ray incident beam size was defined by a 0.2 mm collimator. The system was configured with a VANTEC-2000 2D area detector. The sample to detector distance was 15 cm. The resolution of the detector was 2048×2048 pixels. The aragonite (012) pole figure was collected with the *Φ* rotation. The step size of *Φ* angle is 5° and data collection was done with *Φ*-scan mode. A total of 72 frames were collected to cover the full *Φ* rotation. The pole figure simulation was performed with Multex area software.

## Figures and Tables

**Figure 1:**
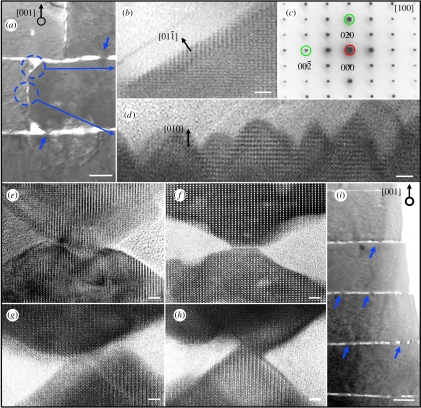
Cross section TEM images demonstrate growth front of aragonite platelets in both vertical [001] and horizontal [010] directions. The nano-structures on the vertical (010) faces of platelets, as marked in (*a*), are magnified in (*b*) and (*d*) where (*c*) is the corresponding selected-area diffraction pattern showing the crystal orientation. (*e*–*h*) demonstrate that [001] outgrowths of nano-asperities from the top and bottom platelets are not exactly connected or epitaxial, even though they share the same crystal orientation as indicated by atomic lattice alignments. The atomic image in (*d*) reveals that those nano-growths are approximately 10–15 nm long and exhibit a larger aspect ratio than the growth structures found on the (001) faces of platelets. Scale bar, 3 nm in (*b*,*d*–*h*) and 200 nm in (*a*,*i*).

**Figure 2:**
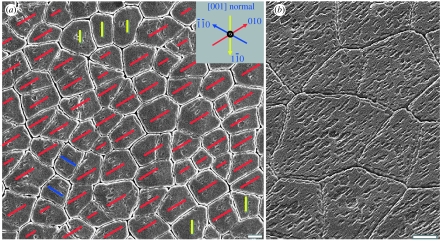
SEM images showing that the imprints of nano-asperity grooves correspond to the crystal directions of the aragonite platelets. (*a*) Exactly three crystal orientations are observed and marked according to the inset. (*b*) A closer view at 35° tilt of the well-aligned nano-asperity imprints. Scale bar, 2 μm.

**Figure 3:**
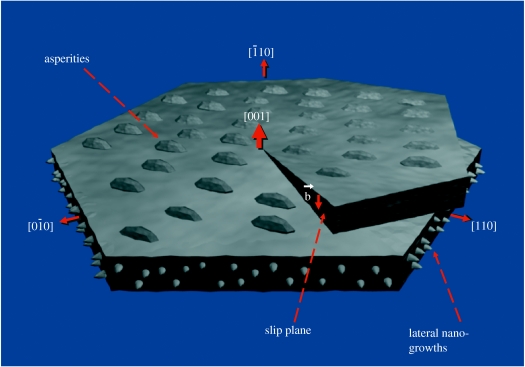
A schematic of the crystallographic directions of an aragonite platelet without surrounding organic layers at a screw dislocation core and the corresponding nano-structures. Asperities, previously interpreted as mineral bridges in lower resolution images, are squat structures found on the [001] faces, while the newly observed [010] or [110] nano-growths are in the vertical plane and have a larger aspect ratio. Note the crystallographic alignment of the asperities, which indicates the platelet crystal orientation. The dislocation slip plane and Burgers vector are labelled.

**Figure 4:**
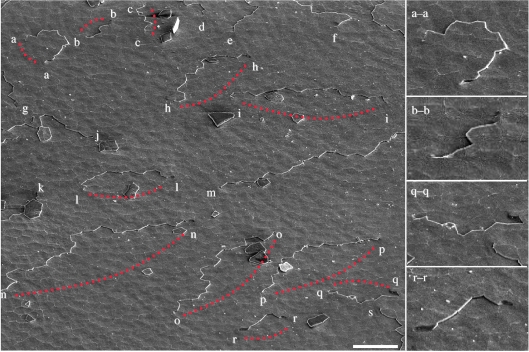
Thirty screw dislocation cores are marked in this low-magnification SEM image. It shows the prevalence of screw dislocation core pairing—i.e. opposite sign (clockwise and anticlockwise) cores share the same growth step, which terminates at both ends at a core. Of the 30 cores, 22 are visibly paired in this image and 4 of them are shown enlarged on the right-hand side. Scale bar, 20 μm.

**Figure 5:**
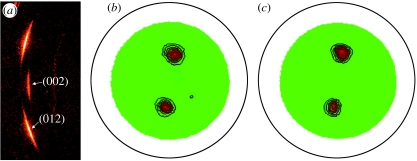
(*a*) X-ray diffraction pattern and (*b*,*c*) pole figure texture measurements of aragonite platelets. The (002) reflection shows that the platelet surface is normal to the vertical growth direction, while the (012) reflection is used for pole figure measurement to probe the lateral orientation of aragonite platelets. The similar features presented in pole figures with both (*b*) 5° and (*c*) 10° incidence angles suggest that the preferred lateral orientation does not change with the X-ray penetration depth.

**Figure 6:**
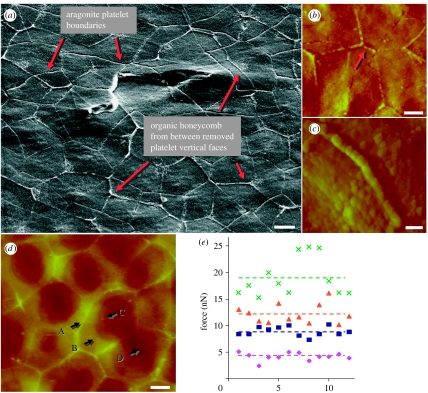
(*a*) SEM image of the exposed nacre surface, revealing that organic layers from between the vertical faces of the removed aragonite platelets remain and form honeycomb-like outlines, effectively allowing the imaging of platelet boundaries from two adjacent layers at once. Two paired screw dislocation cores can also be seen. (*b*) AFM images showing honeycomb-like outlines similar to those in (*a*), revealing in (*c*) that the outline wall consists of a single layer of molecules with a diameter significantly larger than those in the surrounding matrix, and (*d*) demonstrating four surface layers classified by height, marked as A, border; B, top layer; C, middle layer; and D, bottom layer. (*e*) A comparison of experimental data of critical pulling force collected at 12 different locations in those four regions (scale bar, 2 μm in (*a*,*b*,*d*) and 500 nm in (*c*)). Diamonds, border; squares, mid layer; triangles, top layer; crosses, bottom layer; pink dashed line, border average; blue dashed line, mid-layer average; orange dashed line, top layer average; green dashed line, bottom layer average.

**Figure 7:**
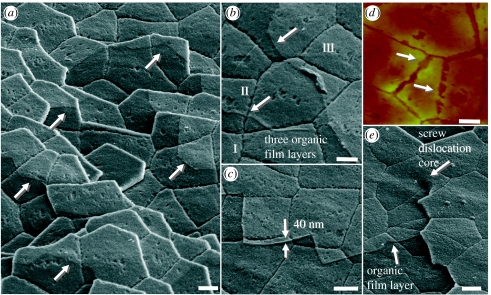
(*a*–*c*,*e*) SEM images show that film-like layers of organic matrix exist throughout all layers of the nacre. Film layer boundaries of various platelets are marked with arrows. Three layers (I–III) ascending from bottom left to top right can be seen clearly in (*b*) and a layer of 40 nm thickness is measured in (*c*). Film-like layers and nano-asperity imprints are also clearly revealed in (*d*) AFM image, and the screw dislocation core in (*e*) reveals that the organic matrix layers follow the spiral from one layer of platelets to the next. Scale bar, 2 μm.

**Figure 8:**
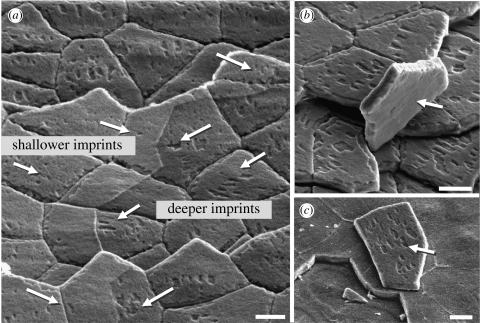
(*a*) SEM images showing various depths of the nano-asperity imprints in the film-like organic matrix layers and (*b*,*c*) differences in intact organic film morphology on both sides of overturned platelets. Scale bar, 2 μm.

**Figure 9:**
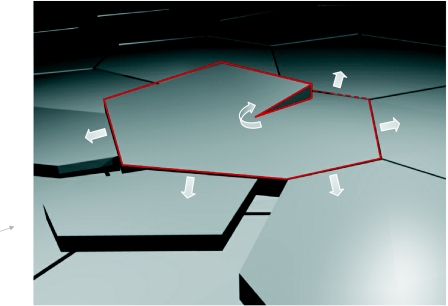
Schematic of the spiral growth model that accounts for both horizontal growth of aragonite sheets and vertical propagation via helices that surround the screw dislocation core.
